# Urolithiasis risk: a comparison between healthcare providers and the general population

**DOI:** 10.1186/s12913-016-1539-7

**Published:** 2016-07-18

**Authors:** Ming-Hung Chen, Shih-Feng Weng, Chien-Chin Hsu, Hung-Jung Lin, Shih-Bin Su, Jhi-Joung Wang, How-Ran Guo, Chien-Cheng Huang

**Affiliations:** Department of Emergency Medicine, Chi-Mei Medical Center, Yongkang District Tainan City, 710 Taiwan; Department of Healthcare Administration and Medical Informatics, Kaohsiung Medical University, Kaohsiung, Taiwan; Department of Biotechnology, Southern Taiwan University of Science and Technology, Tainan, Taiwan; Department of Emergency Medicine, Taipei Medical University, Taipei, Taiwan; Department of Leisure, Recreation and Tourism Management, Southern Taiwan University of Science and Technology, Tainan, Taiwan; Department of Occupational Medicine, Chi-Mei Medical Center, Tainan, Taiwan; Department of Medical Research, Chi Mei Medical Center, Liouying Tainan, Taiwan; Department of Environmental and Occupational Health, College of Medicine, National Cheng Kung University, Tainan, Taiwan; Department of Occupational and Environmental Medicine, National Cheng Kung University Hospital, Tainan, Taiwan; Bachelor Program of Senior Service, Southern Taiwan University of Science and Technology, Tainan, Taiwan; Department of Geriatrics and Gerontology, Chi-Mei Medical Center, Tainan, Taiwan

**Keywords:** Healthcare provider, Nurse, Pharmacist, Physician, Urolithiasis, Specialty

## Abstract

**Background:**

Healthcare providers have many health-related risk factors that might contribute to urolithiasis: a heavy workload, a stressful workplace, and an unhealthy quality of life. However, the urolithiasis risk in healthcare providers is not clear.

**Methods:**

Using Taiwan’s National Health Insurance Research Database, we identified 50,226 physicians, 20,677 pharmacists, 122,357 nurses, and 25,059 other healthcare providers as the study cohort and then randomly selected an identical number of patients who are not healthcare providers (general population) as the comparison cohort for this study. Conditional logistical regression analysis was used to compare the urolithiasis risk between healthcare providers and comparisons. Physician specialty subgroups were also analyzed.

**Results:**

Physicians had a lower urolithiasis risk than did the general population (adjusted odds ratio [AOR]: 0.682; 95 % confidence interval [CI]: 0.634–0.732) and other healthcare providers (AOR: 0.661; 95 % CI 0.588–0.742) after adjusting for hypertension, diabetes, hyperlipidemia, coronary artery disease, and residence location. For pharmacists, nurses, and other healthcare providers, the urolithiasis risk was not significantly different than that for general population. Subgroup analysis showed that surgeons and family medicine physicians had a lower urolithiasis risk than did physician comparisons (AOR: 0.778; 95 % CI: 0.630–0.962 and AOR: 0.737; 95 % CI: 0.564–0.962, respectively).

**Conclusions:**

Although job stress and heavy workloads affect physicians’ health, physicians had a lower urolithiasis risk than did the general population and other healthcare providers. This might be attributable to their greater medical knowledge and access to healthcare. Our findings provide useful information for public health policy makers about the disease risks of healthcare providers.

## Background

Urolithiasis is a common disease with a significant healthcare burden worldwide, especially in a working-age population [1]. Epidemiological studies show that the prevalence rate ranged between 4 %–20 % in developed countries [1, 2]. Because of the improvement of diagnostic procedures and changes in nutrition and environment, the prevalence of urolithiasis is still increasing [3]. Risk factors for urolithiasis are obesity, weight gain, being 50–60 years old, limited fluid intake, diabetes, coronary artery disease, hypertension, and dietary and lifestyle factors (e.g., stress level) [3–6].

Many studies have also shown that some occupations, for example, glass-plant machinists [7], steel workers [8], operating room physicians [9], and naval engineering room workers [10], might predispose individuals to develop urolithiasis; high temperatures and perspiration, for example, might cause dehydration, which would predispose some people to develop urolithiasis.

The risk of urolithiasis in healthcare providers (physicians, pharmacists, nurses, technicians, dieticians, rehabilitation therapists, and social workers) is still unclear. Physicians in particular [11], however, have a heavy and stressful workload, which is also a risk factor for urolithiasis [6]. The only hospital-based case-control study [9] comparing urolithiasis risk between operating room workers and non-operating room workers that our literature review unearthed showed that operating room workers had a significant higher incidence of urolithiasis than did non-operating room workers (14.6 % vs. 9.7 %, *P* = 0.004), and that operating room physicians had an even higher incidence (17.4 %). In addition to the effects of the workplace, a high body mass index, a prior bowel resection, diabetes, and a family history of urolithiasis were independent predictors [9], but the study did not compare the urolithiasis risk between healthcare providers and other non-healthcare providers. Using “urolithiasis,” “healthcare provider,” and “physician” as key words to search for literature indexed in PubMed and Google Scholar, we also did not find any reports on this topic. Differences between physician specialties were never reported, either. Therefore, we wanted to clarify this point. We hypothesized that healthcare providers have a higher risk of urolithiasis than does the general population because their busy and stressful work schedules limit their workday fluid intake.

## Methods

### Data sources

The Taiwan National Health Insurance Research Database (NHIRD) covers about 99 % of Taiwan’s population (23.3 million in 2015) and is one of the largest and most comprehensive databases in the world [12]. The NHIRD contains encrypted patient identification numbers, ICD-9-CM (International Classification of Diseases, Ninth Revision, Clinical Modification) codes for age, gender, dates of hospital visit and discharge, diagnoses, procedures, and prescribed medications [12]. Information on healthcare providers (residence area, types of employment, hospital level, specialty, licensed date, and encrypted identification number) is also available and can be linked to the aforementioned data. National Health Insurance (NHI) covers all the expenses of urolithiasis, diabetes mellitus (DM), hypertension (HTN), coronary artery disease (CAD), and hyperlipidemia.

### Ethics statement

This study was conceived strictly with the Declaration of Helsinki. The Institutional Review Board (IRB) at Chi-Mei Medical Center approved this study and waived the informed consents from the patients because the dataset used in the present study consists of de-identified data released for academic research. Patients’ rights and welfare were not affected by the waiver of the informed consent.

### Selection of healthcare providers and comparisons (general population)

**Fig. 1:**
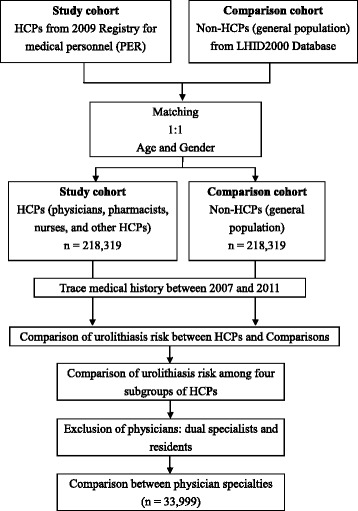
Flow chart for the study. HCP, healthcare provider; LHID, Longitudinal Health Insurance Database

Data of the healthcare providers were obtained from the Registry of Medical Personnel, which contains a record of all registered medical staff in 2009 (Fig. 1). We divided the healthcare providers into four groups for simplicity and similarity: physicians, pharmacists, nurses, and others (technicians, dieticians, rehabilitation therapists, social workers, etc). In the comparison cohort (i.e., general population), we selected from the Longitudinal Health Insurance Database 2000 (LHID2000) one non-healthcare provider match for every healthcare provider case. The LHID2000 contains all the claims data of one million beneficiaries (4.34 % of the total population) randomly selected in 2000. There are no significant differences in age, gender, or healthcare costs between the LHID2000 patients and the NHIRD patients. Comparisons were matched with physicians by age and gender (Fig. 1; Table 1).

**Table 1:** Demographic characteristics and baseline comorbidities for healthcare providers (HCPs) and comparisons (general population) in Taiwan

	Physicians	Comparisons		Pharmacists	Comparisons		Nurses	Comparisons		Other HCPs	Comparisons	
	(*n* = 50,226)	(*n* = 50,226)	*P*	(*n* = 20,677)	(*n* = 20,677)	*P*	(*n* = 122,357)	(*n* = 122,357)	*P*	(*n* = 25,059)	(*n* = 25,059)	*P*
Age (years)												
0–34	12,477 (24.84)	12,477 (24.84)	>0.999	6,062 (29.32)	6,062 (29.32)	> 0.999	76,955 (62.89)	76,955 (62.89)	> 0.999	14,293 (57.40)	14,293 (57.40)	> 0.999
35–59	22,001 (43.80)	22,001 (43.80)		8,358 (40.42)	8,358 (40.42)		38,096 (31.14)	38,096 (31.14)		9,025 (36.02)	9,025 (36.02)	
≥ 60	15,748 (31.35)	15,748 (31.35)		6,257 (30.26)	6,257 (30.26)		7,306 (5.97)	7,306 (5.97)		1,741 (6.95)	1,741 (6.95)	
Mean age in years (SD)												
	44.42 ± 12.16	44.42 ± 12.16	> 0.999	42.89 ± 11.45	42.89 ± 11.45	> 0.999	33.55 ± 8.76	33.55 ± 8.76	> 0.999	34.65 ± 8.78	34.65 ± 8.78	> 0.999
Gender												
Female	9,263 (18.44)	9,263 (18.44)	> 0.999	9,301 (44.98)	9,301 (44.98)	> 0.999	121,096 (98.97)	121,096 (98.97)	> 0.999	16,921 (67.52)	16,921 (67.52)	> 0.999
Male	40,963 (81.56)	40,963 (81.56)		11,376 (55.02)	11,376 (55.02)		1,261 (1.03)	1,261 (1.03)		8,138 (32.48)	8,138 (32.48)	
Baseline Comorbidity												
DM												
Yes	3,530 (7.03)	4,130 (8.22)	< 0.0001	1,308 (6.33)	1,466 (7.09)	0.0019	2,404 (1.96)	2,655 (2.17)	0.0004	569 (2.27)	708 (2.83)	< 0.0001
No	46,696 (92.97)	46,096 (91.78)		19,369 (93.67)	19,211 (92.91)		119,953 (98.04)	119,702 (97.83)		24,490 (97.73)	24,351 (97.17)	
HTN												
Yes	9,742 (19.40)	8,375 (16.67)	< 0.0001	3,313 (16.02)	3,000 (14.51)	< 0.0001	5,554 (4.54)	5,367 (4.39)	0.0671	1,600 (6.38)	1,412 (5.63)	0.0004
No	40,484 (80.60)	41,851 (83.33)		17,364 (83.98)	17,677 (85.49)		116,803 (95.46)	116,990 (95.61)		23,459 (93.62)	23,647 (94.37)	
CAD												
Yes	2,709 (5.39)	2,505 (4.99)	0.0037	875 (4.23)	819 (3.96)	0.1647	1,180 (0.96)	1,224 (1.00)	0.3671	340 (1.36)	314 (1.25)	0.3061
No	47,517 (94.61)	47,721 (95.01)		19,802 (95.77)	19,858 (96.04)		121,177 (99.04)	121,133 (99.00)		24,719 (98.64)	24,745 (98.75)	
Hyperlipidemia												
Yes	8,262 (16.45)	5,583 (11.12)	< 0.0001	2,615 (12.65)	2,171 (10.50)	< 0.0001	6,864 (5.61)	4,189 (3.42)	< 0.0001	1,635 (6.52)	1,069 (4.27)	< 0.0001
No	41,964 (83.55)	44,643 (88.88)		18,062 (87.35)	18,506 (89.50)		115,493 (94.39)	118,168 (96.58)		23,424 (93.48)	23,990 (95.73)	
Residence Location												
North	24,396 (48.57)	26,256 (52.33)	< 0.0001	9,517 (46.03)	11,045 (53.48)	< 0.0001	57,346 (46.87)	65,157 (55.75)	< 0.0001	12,060 (48.13)	13,707 (54.74)	< 0.0001
Center	10,204 (20.32)	8,877 (17.69)		4,120 (19.93)	3,550 (17.19)		22,008 (17.99)	21,520 (17.60)		4,834 (19.29)	4,430 (17.69)	
South	14,468 (28.81)	14,044 (27.99)		6,634 (32.08)	5,648 (27.35)		39,447 (32.24)	30,434 (24.90)		7,519 (30.01)	6,441 (25.72)	
East	1158 (2.31)	994 (1.98)		406 (1.96)	409 (1.98)		3,556 (2.91)	2,135 (1.75)		646 (2.58)	460 (1.84)	

*DM* diabetes mellitus, *HTN* hypertension, *CAD* coronary artery disease. All data are number (%) or mean ± SD

The diagnostic codes were linked through the claims databases of the NHI. Baseline comorbidities that were risk factors for urolithiasis DM (ICD-9 code 250), HTN (ICD-9 codes 401–405), CAD (ICD-9 codes 410–414.02), and hyperlipidemia (ICD-9 code 272). These four comorbidities were counted if they were diagnosed in 3 or more ambulatory care claims including outpatient and emergency visit or one of the inpatient claims coded before January 1, 2009, the index medical care date. In the ambulatory care claims, we used “3 or more” as the criterion in defining ambulatory care claims because in many cases the diagnosis of ambulatory care is not a confirmatory and is for arranging further diagnostic tests. Because we used the same criterion in both study and comparison cohorts, the result should be comparable between two cohorts.

### Comparison between healthcare providers and comparisons (general population)

We compared the urolithiasis risk between the four groups of healthcare providers and comparisons by tracing their medical histories between 2007 and 2011 (Fig. 1). Urolithiasis was identified using the ICD-9 code of 592 or 594.

### Comparisons between healthcare providers, and physician specialty subgroup analyses

We also compared the urolithiasis risk between subgroups of healthcare providers (physicians vs. pharmacists, physicians vs. nurses, etc.) (Fig. 1). Emergency Department and critical care physicians (i.e., internal medicine, surgery, obstetrics and gynecology [Ob/Gyn], pediatrics, and emergency medicine) might have higher stress and workload levels, both of which might contribute to a higher risk for urolithiasis. Thus, we also compared the risk between physician specialties: internal medicine, surgery, Obs/Gyn, pediatrics, emergency medicine, family medicine, and other specialties. Residents were excluded because of their short practice time and dual specialists were excluded (e.g., a physician board certified for internal medicine and emergency medicine) because of the difficulty of assigning them to a specific specialty for analysis.

### Statistical analyses

Differences in baseline characteristics and comorbidities between the groups were evaluated using Student’s *t* test (continuous variables) and Pearson *χ*^2^ tests (categorical variables). We used conditional logistic regression to compare healthcare providers and comparisons and unconditional logistic regression to compare healthcare providers and to compare physician specialties after adjusting for DM, HTN, CAD, hyperlipidemia, and residence location. SAS 9.3.1 for Windows (SAS Institute, Cary, NC, USA) was used for all analyses. Significance was set at *P* < 0.05 (two-tailed).

## Results

### Basic characteristics of healthcare providers and comparisons

We enrolled 50,226 physicians, 20,677 pharmacists, 122,357 nurses, and 25,059 other healthcare providers, and an identical number of age- and gender-matched comparisons (Table 1). Their mean ages were 44.42 ± 12.16 (physicians), 42.89 ± 11.45 (pharmacists), 33.55 ± 8.76 (nurses), and 34.65 ± 8.78 years (other healthcare providers). Most of the physicians (81.56 %) and pharmacists (55.02 %) were men, but most of the nurses (98.97 %) and other healthcare providers (67.52 %) were women. The baseline comorbidity level of DM was significantly lower in all healthcare providers than in comparisons. HTN, however, was significantly higher in physicians, pharmacists, and other healthcare providers, but not in nurses, than in comparisons. CAD was significantly higher only in physicians than in comparisons, but hyperlipidemia was significantly higher in all four subgroups of healthcare providers than in comparisons.

### Comparison of risk for urolithiasis between healthcare providers and comparisons

During a 5-year follow-up period, the period prevalence of urolithiasis in physicians was 2.96 %, in pharmacists was 2.84 %, in nurses was 1.07 %, and in other healthcare providers was 1.76 % (Table 2). Physicians had a significantly lower risk for urolithiasis than did comparisons (adjusted odds ratio [AOR]: 0.682; 95 % confidence interval [CI]: 0.634–0.732) after adjusting for DM, HTN, CAD, hyperlipidemia, and residence location; however, the differences between comparisons and pharmacists, nurses, and other healthcare providers were not significant.

**Table 2:** Conditional logistic regression comparing urolithiasis risk between healthcare providers (HCPs) and comparisons (general population)

	Number (%)	Crude OR (95 % CI)	AOR (95 % CI)
Physicians (*n* = 50,226)	1,485 (2.96)	0.721 (0.673–0.772)**	0.682 (0.634–0.732)*
Comparisons (*n* = 50,226)	2,035 (4.05)	1.00	1.00
Pharmacists (*n* = 20,677)	587 (2.84)	0.996 (0.887–1.120)	0.998 (0.883–1.127)
Comparisons (*n* = 20,677)	589 (2.85)	1.00	1.00
Nurses (*n* = 122,357)	1,304 (1.07)	0.980 (0.907–1.059)	0.983 (0.906–1.067)
Comparisons (*n* = 122,357)	1,330 (1.09)	1.00	1.00
Other HCPs (*n* = 25,059)	442 (1.76)	0.979 (0.858–1.119)	0.956 (0.832–1.098)
Comparisons (*n* = 25,059)	451 (1.80)	1.00	1.00

*AOR* adjusted odds ratio, *DM* diabetes mellitus, *HTN* hypertension, *CAD* coronary artery disease. Adjusted for *DM*, *HTN*, *CAD* hyperlipidemia, and residence location

*P < 0.05; **P < 0.001

### Comparison of risk for urolithiasis between the four subgroups of healthcare providers

When using the other healthcare providers as the reference, physicians also had a significantly lower risk for urolithiasis (AOR: 0.661; 95 % CI: 0.588–0.742). Nurses had a significantly higher risk for urolithiasis (AOR: 1.181; 95 % CI: 1.037–1.346) but no difference after stratification for gender (Tables 3, 4). Both male and female physicians had a significantly lower risk for urolithiasis (AOR: 0.698; 95 % CI: 0.611–0.798 and AOR: 0.584; 95 % CI: 0.433–0.787, respectively) than did other healthcare providers (Table 4).

**Table 3:** Unconditional logistic regression comparing urolithiasis risks among healthcare providers (HCPs)

	Number (%)	Crude OR (95 % CI)	AOR (95 % CI)
Physicians (*n* = 50,226)	1,485 (2.96)	0.589 (0.529–0.656)**	0.661 (0.588–0.742)**
Pharmacists (*n* = 20,677)	587 (2.84)	0.614 (0.542–0.696)**	0.959 (0.842–1.092)
Nurses (*n* = 122,357)	1,304 (1.07)	1.667 (1.495–1.855)**	1.181 (1.037–1.346)*
Other HCPs (*n* = 25,059)	442 (1.76)	1.00	1.00

*AOR* adjusted odds ratio, *DM* diabetes mellitus, *HTN* hypertension, *CAD* coronary artery disease. Adjusted for *DM*, *HTN*, *CAD* hyperlipidemia, and residence location

*P < 0.05; **P < 0.001

**Table 4:** Unconditional logistic regression subgroup analysis by gender comparing urolithiasis risk between healthcare providers (HCPs)

	Number (%)	Crude OR (95 % CI)	AOR (95 % CI)
Males			
Physicians (*n* = 40,963)	1,423 (3.47)	0.970 (0.854–1.103)	0.698 (0.611–0.798)*
Pharmacists (*n* = 9,301)	447 (4.81)	1.361 (1.171–1.583)*	1.002 (0.857–1.170)
Nurses (*n* = 1,261)	40 (3.17)	0.883 (0.631–1.236)	1.185 (0.843–1.666)
Other HCPs (*n* = 8,138)	291 (3.58)	1.00	1.00
Females			
Physicians (*n* = 9,263)	62 (0.67)	0.748 (0.556–1.007)	0.584 (0.433–0.787)*
Pharmacists (*n* = 11,376)	140 (1.23)	1.384 (1.098–1.744)*	0.915 (0.723–1.158)
Nurses (*n* = 121,096)	1,264 (1.04)	1.171 (0.989–1.388)	1.158 (0.976–1.372)
Other HCPs (*n* = 16,921)	151 (0.89)	1.00	1.00

*AOR* adjusted odds ratio, *DM* diabetes mellitus, *HTN* hypertension, *CAD* coronary artery disease. Adjusted for *DM*, *HTN*, *CAD*, hyperlipidemia, and residence location

*P < 0.05

### Comparison of risk for urolithiasis among physician specialties

After excluding residents and dual specialists, we identified 33,999 physicians for the analysis. When using other specialties as the reference, surgeons and family medicine specialists had a significantly lower risk for urolithiasis (AOR: 0.778; 95 % CI: 0.630–0.962 and AOR: 0.737; 95 % CI: 0.564–0.962, respectively); however, internal medicine, Obs/Gyn, pediatrics, and emergency medicine specialists did not have a difference (Table 5).

**Table 5:** Unconditional logistic regression subgroup analysis by physician specialty comparing urolithiasis risk

	Number (%)	Crude OR (95 % CI)	AOR (95 % CI)
Physician specialists			
Internal medicine (*n* = 6,110)	192 (3.14)	1.322 (1.109–1.577)*	0.949 (0.792–1.136)
Surgery (*n* = 4,095)	121 (2.95)	1.241 (1.009–1.527)*	0.778 (0.630–0.962)*
Obs/Gyn (*n* = 1,978)	63 (3.19)	1.341 (1.023–1.758)*	0.914 (0.694–1.204)
Pediatrics (*n* = 2,774)	76 (2.74)	1.148 (0.895–1.474)	0.991 (0.769–1.278)
Emergency medicine (*n* = 479)	8 (1.67)	0.692 (0.342–1.403)	0.762 (0.374–1.551)
Family medicine (*n* = 2,568)	67 (2.61)	1.092 (0.839–1.420)	0.737 (0.564–0.962)*
Other specialties (*n* = 15,995)	383 (2.39)	1.00	1.00

*AOR* adjusted odds ratio, *Obs*/*Gyn* obstetrics and gynecology, *DM* diabetes mellitus, *HTN* hypertension, *CAD* coronary artery disease. Adjusted for *DM*, *HTN*, *CAD* hyperlipidemia, and residence location

*P < 0.05

## Discussion

This nationwide cohort study found, during a 5-year follow-up period, that the period prevalence for urolithiasis was 2.96 % in physicians, 2.84 % in pharmacists, 1.07 % in nurses, and 1.76 % in other healthcare providers. Physicians had a lower urolithiasis risk than the general population; however, pharmacists, nurses, and other healthcare providers did not. When comparing all four subgroups of healthcare providers, physicians had a lower urolithiasis risk than other healthcare providers. In the comparison physician specialties, surgeons and family medicine physicians had a lower risk for urolithiasis than other specialists. This study provided us a new insight about the risk for urolithiasis in healthcare providers. This information might help us make and promote better health policy for the general population.

The possible mechanism that physicians had a lower urolithiasis risk than general population and other healthcare providers may be due to their better medical knowledge, higher disease awareness, and easier healthcare access. In the comparison between physicians and comparisons, we found that physicians had more comorbid HTN, CAD, and hyperlipidemia, which are risk factors for urolithiasis. Physicians were not necessary healthier than the general population; however, their greater medical knowledge and resources may push them take early action to prevent the development of urolithiasis. Chen et al. [13] which compared the risk for acute myocardial infarction (AMI) in physicians and the general population, reported that physicians had a higher prevalence of HTN and hyperlipidemia, but a lower risk for AMI (AOR: 0.57; 95 % CI: 0.46–0.72). Shen et al. [14] also showed that severe sepsis was 24 % less likely to develop in physicians than in the general population. The other reason for the lower risk for urolithiasis is that many physicians self-treat themselves [15, 16].

The reason that emergency medicine and critical care physicians did not have higher risk for urolithiasis might also be because of their greater medical knowledge. Chen et al. [13] reported that physicians who specialized in surgery, internal medicine, emergency medicine, and Ob/Gyn did not have a higher risk for AMI than did other specialists. In spite of more stress, a heavier workload, and greater responsibilities on rotating night shifts, physicians who practice in medical centers had a 58 % lower risk for AMI than did physicians working in local clinics [13]. However, this finding does not mean that overwork did not affect the health of physicians working in medical centers.

Based on the experience of physicians, increasing medical knowledge and resources would substantially benefit everyone in the general population. Whether work- or lifestyle-related, the simplest and most cost-effective way to prevent illness and injury is education [17]. However, both education and sustainable resources (e.g., shelter, food, income) are required to promote and maintain good health [17].

This study has some limitations. First, there was no information on the severity of urolithiasis, family history, obesity, level of fluid intake, diet, metabolic syndrome, social economics status, or lifestyle such as physical activity and exercises; therefore, we were unable to evaluate the effects of these risk factors. Further studies using multivariate logistic regression analysis for adjusting the possible confounding factors above are warranted. Second, the 5-year follow-up period (2007–2011) might not be long enough; thus, additional long-term studies might be needed. Third, although it is possible that the healthy worker effect might confound the results of this study, this effect was minimal because physicians also had a significantly lower risk for urolithiasis than did other healthcare providers. Finally, our findings may not be generalizable to other nations.

## Conclusions

This national population-based cohort study showed the period prevalence of urolithiasis of physicians, pharmacists, nurses, and other healthcare providers in Taiwan during a 5-year follow-up period. Physicians had a higher prevalence of comorbid DM, HTN, and hyperlipidemia but a 32 % lower risk for urolithiasis than did the general population. Comparisons between all four groups of healthcare providers also showed that physicians had a 34 % lower risk for urolithiasis than did other healthcare providers. Surgeons and family medicine physicians had a lower risk for urolithiasis than did other specialists. Physicians specialized in emergency medicine and critical care did not have a higher risk for urolithiasis than did other specialists in spite of their heavier workload. This result implied that other factors such as better medical knowledge and resources might contribute to a lower risk for urolithiasis and that workload did not seem to be major factor for urolithiasis in physicians. Promotion of health education would benefit the public and help the country reduce the economic burden of healthcare.
